# Characterization of genes involved in the iron acquisition system of multidrug-resistant Acinetobacter baumannii

**DOI:** 10.3205/dgkh000480

**Published:** 2024-05-17

**Authors:** Leila Azimi, Hadi Hasani, Abdollah Karimi, Seyed Alireza Fahimzad, Fatemeh Fallah, Shima Fatehi, Shahnaz Armin, Mohammadreza Sadr

**Affiliations:** 1 Pediatric Infections Research Center, Research Institute for Children’s Health, Shahid Beheshti University of Medical Sciences, Tehran, Iran; 2 Department of Medical Surgical Nursing, Jovein School of Nursing, Sabzevar University of Medical Sciences, Sabzevar, Iran; 3 Department of Pediatrics, School of Medicine, Sabzevar University of Medical Sciences, Sabzevar, Iran

**Keywords:** A. baumannii, BauA, BasD, antibiotic resistant

## Abstract

**Background:**

The high prevalence of virulence-associated genes observed in *Acinetobacter baumannii* isolates underscores the pathogenic potential of this bacterium. The presence of these genes confers enhanced survival, evasion of host defenses, and increased virulence. In this study, we investigate the presence and distribution of genes associated with virulence and assess the antimicrobial susceptibility patterns in clinical isolates of *A. baumannii*.

**Materials and method:**

This research focused on examining the 50 multi-drugs resistant (MDR) strains that were included in this investigation. The identification of these strains was validated using Oxa-51. The presence of the *BauA* and *BasD* genes was determined through conventional PCR techniques.

**Results:**

The results derived from Oxa-51 PCR confirmed the identification of all 50 selected strains of *A. baumannii*. Additionally, both the *BauA* and *BasD* genes were successfully identified in 82% of the MDR strains.

**Conclusion:**

Moreover, the varying antibiotic resistance patterns highlight the challenge in treating *A. baumannii* infections effectively. Strategies such as combination therapy, antimicrobial stewardship, and infection control measures should be considered to combat this multidrug-resistant pathogen.

## Zusammenfassung

**Hintergrund:**

Die hohe Prävalenz virulenzassoziierter Gene bei *Acinetobacter baumannii*-Isolaten unterstreicht das pathogene Potenzial dieses Bakteriums. Das Vorhandensein der Gene führt zu verbessertem Überleben, Umgehung der Wirtsabwehr und erhöhter Virulenz. In dieser Studie wurden bei klinischen Isolaten von *A. baumannii* das Vorhandensein und die Verteilung von Genen, die mit Virulenz assoziiert sind, untersucht und die antimikrobielle Empfindlichkeit bewertet.

**Material und Methode:**

Es wurden 50 multiresistente Stämme untersucht. Die Identifizierung wurde mit Oxa-51 validiert. Das Vorhandensein der BauA- und BasD-Gene wurde mit Hilfe herkömmlicher PCR-Techniken bestimmt.

**Ergebnisse:**

Mittels Oxa-51-PCR wurden alle 50 Stämme bestätigt. Darüber hinaus wurden sowohl das BauA- als auch das BasD-Gen in 82% der multiresistenten Stämme identifiziert.

**Schlussfolgerung:**

Die unterschiedlichen Antibiotikaresistenzmuster verdeutlichen die Herausforderung zur wirksamen Behandlung von *A. baumannii*-Infektionen. Zur Bekämpfung dieses multiresistenten Erregers sollten Strategien wie Kombinationstherapie, Antibiotic Stewardship und Maßnahmen zur Infektionskontrolle in Betracht gezogen werden.

## Introduction

*Acinetobacter baumannii* is a Gram-negative bacterium that has become a major concern in healthcare settings due to its ability to cause infections that are difficult to treat [1], [2], [3]. One of the key factors that contribute to its pathogenicity is its iron acquisition system, which allows the bacterium to obtain iron, an essential nutrient for bacterial growth and survival from its host [4], [5]. Understanding the mechanisms and regulation of this system is crucial for developing effective strategies to fight *A. baumannii *infections [4], [5]. The iron acquisition system in *A. baumannii* contributes to its pathogenicity by enabling the bacterium to proliferate and survive within the host [5], [6]. The ability to acquire iron from the host provides *A. baumannii* with a competitive advantage over other bacteria, allowing it to establish infections and evade the host’s immune responses [7]. *A. baumannii* employs several mechanisms to acquire iron from its environment, e.g., siderophores [7], iron-regulated outer membrane proteins (IROMPs), and Heme uptake systems [8]. On the other hand, *A. baumannii* infections caused by antibiotic resistant strains are increasing and making treatment a challenge [9], [10]. 

Understanding the molecular mechanisms of *A. baumannii* and its antibiotic resistance is crucial for developing effective treatment strategies [9]. Two important genes, *BauA* and *BasD*, have been identified in *A. baumannii* and are believed to contribute to its virulence and antibiotic resistance [4], [5]. The *BauA* gene encodes a protein responsible for the binding and uptake of ferric acinetobactin, a siderophore involved in iron acquisition [4]. This allows the bacterium to absorb iron from the host environment, thereby promoting its survival. Additionally, the* BauA* protein has been implicated in biofilm formation, a crucial factor in the persistence and resistance of *A. baumannii* infections. By adhering to surfaces and forming biofilms, the bacterium can evade the immune system and resist antibiotic treatment [4], [11]. The *BasD* gene codes for an enzyme involved in the production of acinetobactin, the aforementioned siderophore. Acinetobactin plays a vital role in the acquisition of iron. The activity of *BasD* is essential for the bacterium’s ability to produce acinetobactin, thereby enhancing its virulence and resistance. Understanding the mechanisms underlying the regulation of *BasD* expression could potentially lead to the development of new therapeutic targets for combating *A. baumannii* infections [5].

Indeed, the prevalence of antibiotic resistance in *BauA* and *BasD* positive strains were significantly greater compared to the equivalent susceptible isolates [5], [12]; even the antibiotic cross-resistant profile is found more often in MDR *A. baumannii* isolates which possess some virulence genes, such as *BauA* [5]. This indicates that drug-resistant *A. baumannii* isolates seem to possess enhanced toxicity [5], making the identification of *A.baumannii* with virulence genes such as *BauA* and *BasD* is necessary. Hence, this study aimed to determine the prevalence and frequency of *BauA *and* BasD* genes in multi-drug resistant strains of *A. baumannii*.

## Materials and methods

This detailed analysis, conducted as part of a research study, included a total of 50 multidrug-resistant (MDR) *A. baumannii* strains. These specific varieties were gathered from various divisions within ten educational medical facilities situated in Iran.

The considered isolates studied here are associated with various units within the hospital, such as the intensive care unit (ICU), surgical department, neonatal intensive care unit (NICU), and others. Furthermore, there were instances where bacterially caused infections originated from different sources, e.g., blood, urine, and wounds.

Initially, the confirmation of *A. baumannii* was achieved by amplifying the Oxa-51 gene using specific forward and reverse primers, 5'-TAATGCTTTGATCGGCCTTG-3' and 5'-TGGATTGCACTTCATCTTGG-3', respectively [3]. The identification of the target genes was then performed through conventional PCR, under previously established experimental conditions. For DNA extraction, a boiling method was employed, and the extracted DNA samples were stored at –80ºC until the PCR analysis was conducted. The following primers were used for simultaneous gene duplication of *BauA* and *BasD* genes according to the results of the primer BLAST. NCBI (National Center for Biotechnology Information) [12]. These primers possess the ability to detect and determine the presence of both the *BauA* and *BasD* genes, simultaneously. The primers sequencing are; Forward: 5'-CTCTTGCATGGCAACACCAC-3' and Reverse: 5'-CCAACGAGACCGCTTATGGT-3' [5], [13].

## Results

Our results indicate that the majority of these isolates were collected from the ICU. In terms of prevalence in this study, invasive catheters were commonly linked to these bacterial infections, followed closely by blood culture.

The identification of all *A. baumannii* was confirmed according to the results of the PCR for the Oxa-51 gene. Moreover, the results of PCR and gel electrophoresis showed that in 82% of the MDR strains, both *BauA* and *BasD* genes were successfully detected.

## Discussion

Iron acquisition is a crucial aspect of *A. baumannii* pathogenesis, enabling the bacterium to survive and cause infections in the host [4], [5]. Understanding the mechanisms and regulation of iron acquisition in *A. baumannii* is essential for the development of effective therapeutic strategies. Further research in this field will provide valuable insights for combating *A. baumannii* infections and reducing their impact on healthcare settings [7], [13]. The *BauA* and *BasD* genes in *A. baumannii* play crucial roles in promoting the bacterium’s virulence and antibiotic resistance [5], [13]. The *BauA* protein facilitates iron acquisition and biofilm formation, while the *BasD* enzyme is responsible for the production of acinetobactin. Both genes contribute to the bacterium’s ability to survive and cause persistent infections [5], [10]. Further research is needed to fully elucidate the mechanisms by which *BauA* and *BasD* influence antibiotic resistance, thus providing valuable insights for the development of effective treatment strategies against *A. baumannii* infections. 

In the present study, *BauA* and *BasD* genes in MDR *A. baumannii* were present at a rate of 82%. Conversely, a study conducted in Iran by Porbaran et al [4] revealed a lower frequency of 15.2% and 12.5% for the *BauA* and *BasD* genes, respectively in *A. baumannii*. This disparity in frequencies could be attributed to differences in strain selection between the two studies. In the current study, MDR *A. baumannii* strains were chosen, while Porbaran et al. [4] selected *A. baumannii* strains with different patterns of antibiotic resistance. The *BauA* gene serves as one of the mechanisms for antibiotic resistance in *A. baumannii*, and it is evident that its frequency is higher in MDR strains.

In China in 2018, the *BauA *and* BasD* genes were found to have a frequency of 78.3% and 95.7%, respectively in the MDR *A. baumannii* strain [5]. The outcomes of that particular investigation [5] are closely comparable to those of the present study, since both studies focused on MDR strains. Additionally, it reinforces the notion that the presence of the *BauA* gene leads to antibiotic resistance [4].

Porbaran et al. [4] also revealed a noteworthy correlation between the distribution of iron/siderophore-uptake genes (e.g., *BauA*) and antibiotic resistance. Furthermore, another study demonstrated a high frequency of the gene encoding *BauA*, particularly within multidrug-resistant isolates [13]. This finding aligns perfectly with our data. The frequency of *BauA* and *BasD* genes was high in MDR *A. baumannii* which was included in this study.

## Conclusion

This study provides valuable insights into the frequency of *BauA* and *BasD* genes in *A. baumannii* clinical isolates. The high prevalence of these genes emphasizes the need for enhanced surveillance and infection control measures to limit the spread of multidrug-resistant *A. baumannii* strains. Regional differences in gene frequencies indicate the importance of tailored intervention strategies based on the specific resistance mechanisms prevalent in different healthcare settings. Further research is warranted to explore the clinical implications of these findings and to develop effective strategies to mitigate the impact of *A. baumannii* antibiotic resistance.

## Notes

### Funding

The research reported in this publication was supported by Elite Researcher Grant Committee under grant number [401201] from the School of Medicine, Sabzevar University of Medical Sciences, Sabzevar, Iran.

### Ethical approval

The ethical approval number of this study is “IR.MEDSAB.REC.1401.110” from the School of Medicine, Sabzevar University of Medical Sciences, Sabzevar, Iran. 

### Acknowledgements

We would like to thank the Pediatric Infections Research Center, Research Institute for Children’s Health, Shahid Beheshti University of Medical Sciences, Tehran, Iran, for performing the laboratory tests and storing samples, along with the Cellular and Molecular Research Center, Sabzevar University of Medical Sciences, Sabzevar, Iran for their scientific support of the research.

### Authors’ ORCID

- Leila Azimi: 0000-0002-7216-2530
- Hadi Hasani: 0000-0002-3070-3108
- Abdollah Karimi: 0000-0002-4225-0097
- Seyed Alireza Fahimzad: 0000-0001-6054-0656
- Fatemeh Fallah: 0000-0002-3380-9549
- Shima Fatehi: 0009-0005-0914-6078
- Shahnaz Armin: 0000-0002-4993-482X
- Mohammadreza Sadr: 0000-0001-5376-0933

### Competing interests

The authors declare that they have no competing interests.
